# Reliability of a Novel Preoperative Protocol for Determining Graft Sizes for Superior Capsular Reconstruction Using Plain Film Radiography

**DOI:** 10.3390/jcm12072707

**Published:** 2023-04-04

**Authors:** Ryan S. Ting, Ron Rosenthal, Tsz Kit Law, Hilal S. A. Al-Housni, Lisa Hackett, Patrick H. Lam, George A. C. Murrell

**Affiliations:** Orthopaedic Research Institute, St George Hospital Campus, University of New South Wales, Sydney, NSW 2217, Australia

**Keywords:** superior capsule reconstruction, irreparable rotator cuff tear, synthetic, PTFE, Teflon

## Abstract

Background: Superior capsular reconstruction (SCR) for massive, irreparable rotator cuff tears involves anchoring a graft between the superior glenoid and the greater tuberosity of the humerus. Optimizing the graft size is important. We aimed (1) to evaluate the reliability of plain film radiography in determining graft size for SCR and (2) to create a database to help predict future graft sizes. Methods: An inter- and intra-rater reliability trial was conducted on 10 and 6 subjects with healthy shoulders, respectively, using plain film radiography to measure the distance between the superior glenoid and the supraspinatus footprint. The subjects were positioned upright with an abduction pillow modified to hold the shoulder at 30° abduction and 45° external rotation, afterwhich a true antero-posterior shoulder radiograph was captured. Thirty subjects were recruited for the database and grouped using the aforementioned protocol. Results: The inter-rater and intra-rater trial agreement was excellent, with intraclass correlation coefficients of 0.94 (95% CI) and 0.76 (95% CI), respectively. Three medio-lateral patch sizes, of 33 mm, 38 mm, and 47 mm, were proposed based on the protocol in 30 subjects. Conclusions: Plain film radiography demonstrated excellent reliability in measuring the distance between the superior glenoid and the supraspinatus footprint. Three ordinal patch sizes are proposed.

## 1. Introduction

Massive, irreparable rotator cuff tears are difficult injuries to treat. They are characterized by the retraction of the torn supraspinatus tendon to an extent where it can no longer reach its native footprint on the greater tuberosity of the humerus. There are a number of approaches to the operative management of such tears, including arthroscopic debridement, interposition with synthetic [1,2,3,4,5] or biological [6,7,8] grafts, tendon transfers [9,10,11], and reverse total shoulder arthroplasty [12,13,14].

Whilst the aforementioned strategies have their merits, Mihata et al.’s SCR is a relatively novel joint-preserving treatment that does not rely on the torn tendon as interposition grafts do, and may be particularly beneficial for younger patients who are too young for arthroplasty [15,16]. Fascia lata was the original graft material used. However, biomechanical studies have found human dermal allograft and synthetic polytetrafluoroethylene (PTFE) provide comparable structural properties [17,18,19]. Furthermore, patch grafts circumvent the need to harvest autografts and the associated donor site morbidity [17,20,21].

The size of the patch is important for the success of this procedure. Mihata’s protocol described as optimal anteroposterior length, exactly the length of the defect, whereas optimal mediolateral length was 15 mm longer than the distance from the superior glenoid to the supraspinatus insertion point. This excess accommodated for the creation of a 15 mm patch footprint on the superior glenoid [22,23,24].

We hypothesized that our preoperative protocol for the measurement of the mediolateral length using plain film radiography in a standardized position could provide valuable information to guide the surgeon intraoperatively. A further development of this concept was the creation of a patient database to propose ordinal patch sizes that would be appropriate for patients within a range.

To our knowledge, there has been no study to provide a standardized preoperative protocol using plain film radiography to measure the distance between the superior glenoid and the supraspinatus insertion point for superior capsular reconstruction. The aims of the present study were therefore (1) to evaluate inter-rater and intra-rater reliability in measuring the distance between the superior glenoid and the supraspinatus insertion point, and (2) to apply this a preoperative protocol to the creation of a database for determining the appropriate mediolateral dimension of synthetic patch grafts for SCR.

## 2. Materials and Methods

Ten subjects with normal shoulders were recruited for this study. An inter- and intra-rater reliability trial was conducted using plain film radiography to assess the distance between the superior glenoid and the supraspinatus insertion point on the greater tuberosity of the humerus. Thirty subjects were recruited for the creation of a database to propose three standardized patch sizes for future operations.

This research was approved by the IRB of the authors’ affiliated institutions. The study was conducted according to the guidelines of the Declaration of Helsinki and approved by the Ethics Committee of the South Eastern Sydney Local Health District (LNR/13/POWH/186 on 1 May 2022). Informed consent for enrolment into the study and for the publication of these findings was obtained from each participant.

The inclusion criterion for the inter- and intra-rater reliability trial and the database component of the study was subjects with shoulders capable of a minimum of 30° passive abduction and a minimum of 45° passive external rotation. The exclusion criteria for both the inter-and intra-rater reliability trial and the database component of the study were: (1) <18 years of age, (2) glenohumeral arthritis, (3) superior migration of the humeral head, (4) previous rotator cuff repair, and (5) previous shoulder arthroplasty.

### 2.1. Equipment

A Carestream ODYSSEY HF Series^TM^ (Quantum Medical Imaging, North Ronkonkoma, NY, USA) X-ray machine was used for all examinations.

### 2.2. Raters

Three raters participated in this study: Rater A, a musculoskeletal radiographer with over 20 years of experience, Rater B a medical student with no experience in plain film radiography, and Rater C, a medical practitioner with limited experience in plain film radiography.

All raters were briefed on the study protocol. A training session was held to ensure positioning, imaging technique, application of the Carestream system, and landmarks for the placement of the calibration and ruler markers were standardized for accurate measurement.

### 2.3. Positioning Protocol

All subjects were examined standing upright, with the shoulder of interest placed at 30° of abduction and 45° of external rotation. To create 30° of abduction, an abduction pillow was placed above their ipsilateral iliac crest and secured with a waist strap. To create 45° of external rotation, a foam pad was placed between the abduction pillow and the subject’s forearm, whilst keeping the subject’s elbow flush against the abduction pillow.

**Figure 1:**
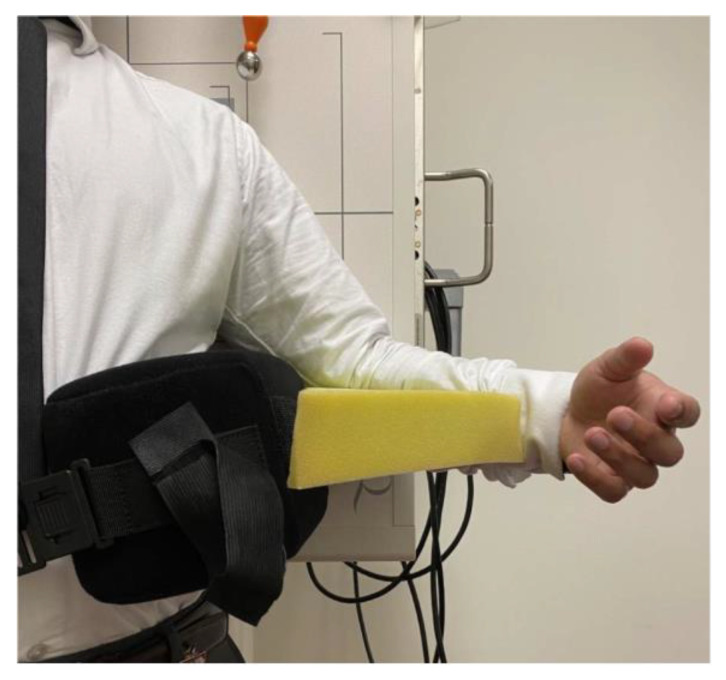
Subject positioning with abduction pillow (black) and foam pad (yellow).

A true anterior-posterior view of the glenohumeral joint was used for the evaluation. Thus, the subject’s scapula on the imaged side was laid flush against the image detector. As a result, the subject’s thorax was rotated toward the affected shoulder at 30–45°. (Figure 1).

### 2.4. Scanning Protocol

To ensure standardization, the X-ray tube was positioned at 20° caudal angulation centered at the glenohumeral joint. The superior glenoid and greater tuberosity were used as reference landmarks for measurement and quality of the image. A radiographic reference ball was placed at the level of the coracoid process, in contact with the subject’s skin.

**Figure 2:**
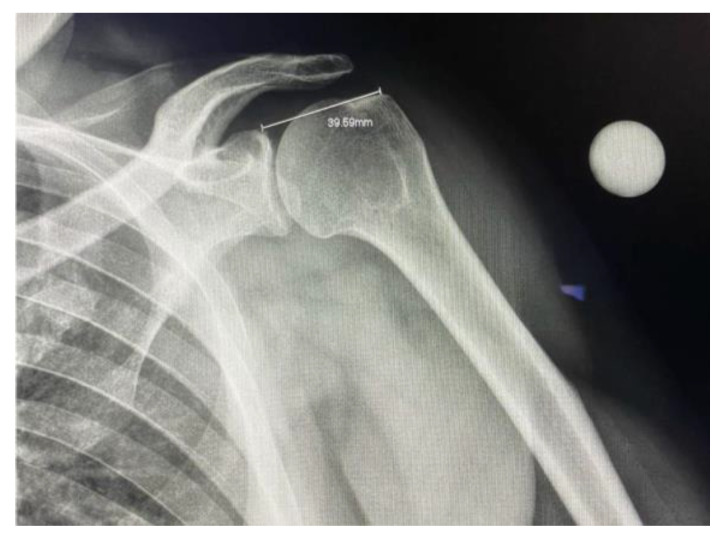
Plain film radiograph of the glenohumeral joint with a measurement of the distance between the superior glenoid and the insertion point of supraspinatus.

After capturing the plain film radiograph, the raters were instructed to calibrate the system using the radiographic reference ball. Using the calibration measurement tool, a straight line was drawn through the diameter of the ball, and a real length value of 25 mm was entered manually. Upon calibration, the ruler device was used to place a marker at the superior aspect of the glenoid where the medial anchors would be placed and another at the greater tuberosity where the lateral anchors would be placed. The distance would then be generated. (Figure 2).

### 2.5. Inter-Rater Trial

For the evaluation of inter-rater reliability, the ten subjects were examined by the three raters. Each rater positioned each subject and captured three different radiographs of the same subject with a 5 min interval between capturing each radiograph. A total of 90 assessments were performed for the inter-rater trial. The subject was repositioned at the beginning of each inter-rater trial.

### 2.6. Intra-Rater Trial

For the evaluation of intra-rater reliability, Rater A examined six subjects, obtaining three radiographs per examination. Therefore, each subject was evaluated three times, with a one-hour interval between each assessment. A total of 18 assessments were performed for the intra-rater trial. The subject was repositioned at the beginning of each assessment in accordance with the aforementioned protocol. Rater A positioned, captured, and assessed the radiographs independently of external inputs from other raters.

### 2.7. SCR Database

A cohort of 30 subjects who presented to the clinic were recruited. A database for recording the distance between the superior glenoid and the supraspinatus insertion point on the greater tuberosity of the humerus using the aforementioned protocol was generated. The results were measured and grouped using a histogram to propose patch sizes for SCR.

### 2.8. Statistical Analysis

The reliability of plain film radiography in calculating the distance between the superior glenoid and the greater tuberosity was assessed with a two-way random-effects model to generate interclass correlation coefficients (ICC) with 95% confidence intervals, using SPSS version 24. ICCs were interpreted with the guidelines formulated by Cicchetti and Sparrow: 0.00 to 0.39, poor; 0.40 to 0.59, fair; 0.60 to 0.74, good; 0.75 to 1.00, excellent [25].

## 3. Results

The 10 subjects recruited for the inter-rater trial comprised 4 females and 6 males. The 30 subjects recruited for the database comprised 16 males and 14 females.

### 3.1. Inter-Rater Testing

The reliability of the measurement between the superior glenoid and the point of insertion of the supraspinatus in the inter-rater trial was excellent. The ICC value was 0.94 (95% CI), with a lower bound value of 0.84 and an upper bound value of 0.98. The average measurement of the space was 26.5 mm, with a standard deviation of 0.7 mm between the raters.

### 3.2. Intra-Rater Testing

The reliability of the measurement between the superior glenoid and the point of insertion of the supraspinatus in the intra-rater trial was excellent. The ICC value was 0.76 (95% CI), with a lower bound value of 0.34 and an upper bound value of 0.96. The average measurement of the space was 25.9 mm, with a standard deviation of 0.7 mm between each session.

### 3.3. Database Result

**Figure 3:**
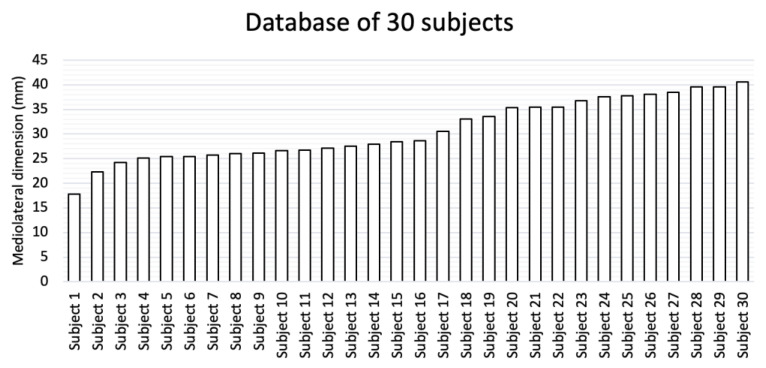
Database of the mediolateral dimension between the superior glenoid and the point of insertion of supraspinatus.

**Figure 4:**
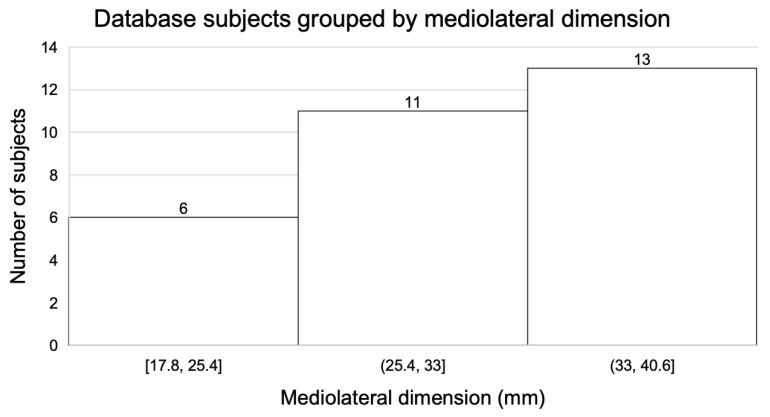
Database subjects, grouped by the mediolateral dimension between the superior glenoid and the point of insertion of the supraspinatus.

A database of 30 recorded measurements between the superior glenoid and the point of insertion of the supraspinatus using our X-ray protocol was established (Figure 3). The subjects were then allocated to one of three groups (Figure 4) based on the mediolateral dimensions generated by the protocol. The dimensions of the six subjects in the first group ranged from 17.8 mm to 25.4 mm, with a mean of 23 mm. The dimensions of the eleven subjects in the second group ranged from 25.4 mm to 33 mm, with a mean of 28 mm. The dimensions of the thirteen subjects in the third group ranged from 33 to 40.6 mm, with a mean of 37 mm. The mean distance of the database group overall was 30.8 mm and ranged from 17 mm to 40.6 mm, with a standard deviation of 6.2 mm.

To accommodate for the surgical landing sites and areas for suturing both medially and laterally, 10 mm was added to the mean of each of the three groups. Thus, the proposed patch sizes, from small to large, were 33 mm, 38 mm, and 47 mm.

## 4. Discussion

This study investigated the reliability of using plain film radiographs in measuring the distance between the superior glenoid and the point of insertion of the supraspinatus on the greater tuberosity. Statistical analyses demonstrated excellent reliability of this protocol, regardless of the rater experience. A database was generated using the protocol, and three standardized patch sizes were subsequently proposed for use in future superior capsular reconstructions.

Whilst massive, irreparable rotator cuff tears are best visualized using soft tissue imaging modalities such as MRI, the sequelae of cuff tear arthropathy that ends in glenohumeral acetabulization and osteoarthritis mean that plain film radiographs are also often required to assess the extent of bony damage [16,26]. In contrast to MRI, plain film radiography is readily accessible, inexpensive, and timely, both in the conduction of the study and in the development of the images. Whilst ultrasound is also an inexpensive soft-tissue imaging modality, it is user-dependent and dynamic, which in the context of preoperative templating makes it less suitable than a plain film radiograph [27]. Nonetheless, 2D radiographic investigations such as plain film radiographs are subject to variability, with factors including positioning and appropriate choice of landmarks, which are just two of the multiple factors that can influence a result. Thus, a reliability assessment was required to test the robustness of our novel protocol.

To our knowledge, there have been no studies to design a standardized preoperative imaging protocol to template graft sizes in superior capsular reconstruction. Previous technical notes described the use of a measuring probe intraoperatively to determine the dimensions of the graft [22,23,24,28,29]. However, arthroscopy is a highly technical procedure, and as expected, proficiency is gained through experience. Accordingly, intraoperative arthroscopic measurement speed and accuracy is lower for less experienced surgeons [30,31]. Furthermore, given the relatively recent debut of Mihata’s SCR in 2012, a reliable preoperative radiographic protocol would aid in minimizing intraoperative measurement errors. In addition, preoperative planning may help to facilitate the procedure, as increased operative time has been shown to be an independent predictor of surgical site infection and hospital length of stay [32].

There are many graft options available on the market, each varying in area, thickness, and material. These factors invariably influence the cost of the procedure. A greater graft thickness has shown to be an important factor in maintaining the acromiohumeral distance, with superior biomechanical properties compared to those of thinner grafts [20,33]. SCR grafts are commonly folded upon themselves to increase their thickness. Therefore, a preoperative reference on the required area of the graft to bridge the gap between the superior glenoid and the greater tuberosity allows the surgeon to account for the excess required to create the ideal thickness of 6–8 mm [34].

The strength of this study is the ease and reliability with which this protocol can be implemented, independent of the rater experience. One limitation for both components of this study was the cohort size. A limitation of the database component is that our subjects may not be representative of other age or ethnic groups.

## 5. Conclusions

Our preoperative X-ray protocol demonstrated excellent reliability in measuring the distance between the superior glenoid and the insertion of the supraspinatus tendon, independent of the rater experience. The utilization of this easily replicable protocol and its database will provide valuable information for surgeons performing superior capsular reconstruction.

## Data Availability

Available upon request.
